# Lord Dawson's Scheme

**Published:** 1920-06-05

**Authors:** 


### The Hospital

The Workers' Newspaper of Administrative Medicine and Institutional Life, Administration, National Insurance and Health.

No. 1774, Vol. LXVIII. SATURDAY, JUNE 5, 1920. PRICE TWOPENCE

### LORD DAWSON'S SCHEME.

The Consultative Council appointed last year by the Minister of Health and presided over by Lord Dawson of Penn, has presented an interim report containing the provisional conclusions arrived at concerning the organisation of a complete Health Service for the community. In essentials this report adopts the views first publicly advocated by Lord Dawson himself, and entails some very sweeping changes in the whole relations of patients, doctors, and institutions for the sick. The Ministry of Health, and still more the Government, is not committed to what may be called Lord Dawson's scheme; and if they do adopt the general idea, there will be ample opportunities for the discussion of details before it can possibly be translated into law. But it is quite within the bounds of possibility that the report foreshadows the general trend of future events, and the Consultative Council's memorandum is therefore of transcendent importance. An outline of its principal provisions was published in last week's issue (The Hospital, May 29, p. 221), from which it is to be inferred that a form of modified State Medical Service is proposed which shall sweep in practically all the population, all the doctors, and all existing institutions. On the other hand, a definite attempt is made to minimise the bureaucratisation of the health services by maintaining "free choice of doctor," though it is very uncertain how far any such attempt is likely in the long run to succeed. From the point of view of patient and doctor in their domicilian relations, the success of this part of the scheme depends mainly on two factors: first, the prevention of machine-made medicine, scamped service, and the deadening effect upon the individual of service under a State Department (a very real danger, as hundreds of demobilised temporarily commissioned doctors can testify); and, second, the evolution of a much better professionally equipped type of general practitioner than the National Health Insurance panels have yet succeeded in attracting.

From the institutional aspect the scheme is even more far-reaching. The Primary and Secondary Health Centres which are proposed for creation are in reality institutions which must replace all—or almost all—existing nursing homes, cottage hospitals, general and special hospitals; and the latter will consequently either be snuffed out of existence or be absorbed into the State scheme. That this is the case can be seen from the Consultative Council's memorandum, which contemplates that for many Secondary Centres the nucleus of the organisation will be found in existing hospitals; and where these nuclei are not available, it is stated, it will be necessary to establish "complete and model" health centres, which will "need a complete hospital equipment." The first criticism of this ambitious (but not therefore necessarily impossible) scheme, is that the cost of it will be prohibitive; and in the present state of the national finances this is perhaps the gravest objection that the Consultative Council will have to answer. Clearly enough the vast expense entailed will be reduced to a minimum by the process of expropriating the voluntary and the rate-supported hospitals upon which the institutional system of this country at present depends. Whether or not such a revolution is contemplated, whether or not if contemplated it is actually avowed, it is difficult to see how the voluntary system can stand side by side with the State system here adumbrated, as some of the organs of the daily press are suggesting. For one thing, the Health Service will be practically forced into taking over the voluntary hospitals to make its scheme financially possible at all; and for another the springs of private charity are hardly likely to go on flowing as they do at present when those who at present support the voluntary hospitals are enduring additional heavy taxation entailed by the establishment of the State Service. (*See also p.* 253.)

That the proposals of the Consultative Committee are attractive in many respects it is impossible to dispute; but in so far as they entail the disappearance of the voluntary system, they are, therefore, to be scrutinised and considered with the closest attention. It rests with the people themselves to pronounce the verdict. Do they desire the annihilation of the voluntary system of hospitals with the splendid results it has achieved and the new and high standards it has set in medicine, surgery, research, administration, nursing, and all that makes up the voluntary hospital as we know it to-day? We do not believe it. But they must make their position plain and establish beyond doubt their confidence that the voluntary system is essential to the well-being of the sick in this country, and that their intention is, by every means in their power, to oppose its extinction.

